# Improvement of Inhalation Profile of DPI Formulations by Carrier Treatment with Magnesium Stearate

**Published:** 2009

**Authors:** Shruti Hazare, Mala Menon

**Affiliations:** Bombay College of Pharmacy, Kalina, Santacruz (E), Mumbai-400 098, India

**Keywords:** Magnesium stearate, microspheres, dry powder inhalation, catalase

## Abstract

In the present study, the applicability of magnesium stearate-treated lactose carrier particles was evaluated to improve the inhalation performance of catalase microspheres. Bovine liver catalase microspheres with different stabilizers were prepared by spray drying. For the surface modification, lactose products were treated with 1 and 2% magnesium stearate by manual mixing and gently passed through a # 60 size mesh. The results revealed that magnesium stearate can be applied as a performance improver for catalase microsphere based DPI formulations.

Dry powder inhalations (DPI) have become a core area of respiratory drug delivery in which the carrier particles play a critical role in preventing the agglomeration of fine drug particles. To achieve high deposition in deep lungs to satisfy the required bioavailability, the fine drug particles should detach from the coarse carrier particles through inhalation process[1]. A decrease in surface roughness and the surface free energy of carrier particles can improve the aerosol inhalation efficiency of the drug-carrier blend. The use of various agents like L-leucine, magnesium stearate as performance modifiers are important to improve flow, reduce inter-particulate adhesion and reduce moisture effects, and thereby achieve improved particle deposition in lungs[2]. In the present study, the applicability of magnesium stearate-treated lactose carrier particles was evaluated to improve the inhalation performance of catalase microspheres, for the treatment of oxidative stress-induced lung disorders.

## MATERIALS AND METHODS

Bovine liver Catalase (2950 units/mg, Sigma Aldrich) microspheres with different stabilizers (Batches A, B, C) were prepared by spray drying (Labultima LU-222 Advanced laboratory spray drier, India). Particle size distribution of microspheres was estimated by laser diffraction (Malvern, UK- master sizer 2000). For the surface modification, lactose products (Lactohale 200, a gift from Friesland Foods Domo, Netherlands) were treated with 1% and 2% magnesium stearate (S. D. Fine Chem) by manual mixing and gently passed through mesh (60 #). For the preparation of DPI formulation, treated lactose carrier and catalase microspheres (1:6, 1:8) were gently mixed in geometric manner, passed through mesh (60 #) and then filled into Number 3 hard gelatin capsules (Associated Capsules, India). The ability of formulations to aerosolize using Rotahaler® (Cipla Ltd., India) was determined by twin stage impinger (British Pharmacopoeia, 2004) using air at 60 l/min for 5 sec. The inhalation properties of the DPI formulations were evaluated using an Anderson Cascade Impactor (ACI, Copley, U.K.) with an inhalation device Rotahaler® using air flow rate at 60 l/min for 5 sec at 20±2°/40% relative humidity.

## RESULTS AND DISCUSSION

**Fig. 1:**
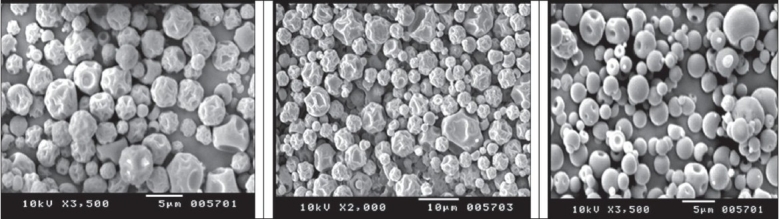
SEM of Microspheres

**Fig. 2:**
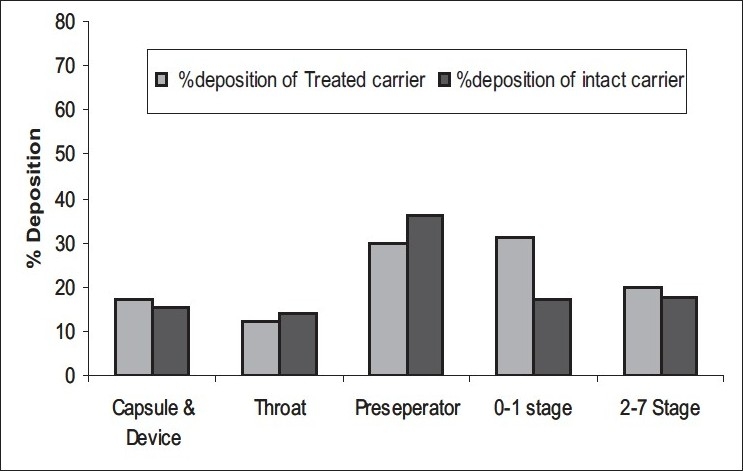
ACI performance of Batch A (1:6)

Microspheres were obtained as buff colored, near spherical particles with corrugated surface (fig. 1). Particle size was found to be 4-5 μm (D_50_). In case of batches A and B TSI data revealed higher fine particle fraction (FPF) and dispersibility with the carrier treated with magnesium stearate compared to that obtained with intact carrier and plain microspheres. Carrier treated with 2% magnesium stearate showed better performance than carrier treated with 1% magnesium stearate. With batch C, 1:6 microspheres:carrier ratio did not show any improvement in FPF but with increase in microspheres:carrier ratio to 1:8, significant improvement in FPF was seen (Table 1). Based on TSI study data batch A (intact carrier and 2 % treated carrier), was further taken up for ACI study. Carrier treated with magnesium stearate was observed to decrease the deposition of microspheres on the upper part, especially on the preseperator, and increased deposition was seen on the lower stages compared to the intact carrier (fig. 2,Table 2). The study reveals the applicability of magnesium stearate as performance improver for catalase microsphere based DPI formulations by covering the high energy binding sites on carrier particles and increasing the dispersibility of microspheres on carrier surface.

**TABLE 1:** TSI PROFILE OF PLAIN MS AND DPI FORMULATIONS

Batches	Plain MS	Intact Carrier	Mg-stearate treated carrier	
			
			1%	2%
**A (1:6)**				
% FPF	25.78±0.41	27.86±0.6	30.63±0.91	36.1±0.74
% Dispersibility	53.19±0.84	36.36±0.78	44.03±1.33	47.06±0.26
**B (1:6)**				
% FPF	15.74±0.89	25.89±0.95	x	30.68±0.46
% Dispersibility	37.43±2.11	37.36±1.37	x	38.52±0.59
**C (1:6)**				
% FPF	x	19.4±0.31	x	19.71±0.51
% Dispersibility	x	36.15±0.57	x	23.73±0.62
**C (1:8)**			
% FPF	x	21.54±0.25	x	30.84±0.82
% Dispersibility	x	25.33±0.45	x	37.97±1.01

Data presented are mean±SD, n=3. %FPF = FPD/RD ×100, Fine Particle Dose (FPD) -Deposition in stage II, RD- Recovered Dose. %Dispersibility = (FPD/ED)×100, ED- Emitted Dose

**TABLE 2:** ACI PROFILE OF BATCH A DPI FORMULATION

Batch A	MMAD±GSD (μm)	% FPF
Intact carrier	3.98±1.33	32.175
Treated carrier	4.03±1.32	38.42

## References

[1] Bisgaard H, O'Callaghan C, Smaldone G Drug Delivery to the Lung, Marcel Dekker.

[2] Kumon M, Machida S, Suzuki M, Kusai A, Yonemochi E, Terada K (2008). Application and Mechanism of Inhalation Profile Improvementof DPI Formulations by Mechanofusion with Mg-Stearate. Chem Pharm Bull.

